# Soil erosion risk assessment and treatment priority classification: A case study on guder watersheds, Abay river basin, Oromia, Ethiopia

**DOI:** 10.1016/j.heliyon.2022.e10183

**Published:** 2022-08-10

**Authors:** Timketa Adula Duguma

**Affiliations:** Department of Agricultural Engineering, Ambo University, Hachalu Hundessa Campus, Institute of Technology, Ambo, P.O.Box 19, Ethiopia

**Keywords:** Guder, Upper blue Nile river, Oromia, USLE, Erosion hazard classification

## Abstract

Soil erosion is the most persistent environmental problem in the Upper Blue Nile River (UBNR) basin of Ethiopia. Guder River is one of thetributaries of UBNR basin which critically required soil conservation practices. The main objective of this particular research article was to appraise soil erosion hazard priority classification with an easy and uncomplicated erosion modelling tool, the universal soil loss equation (USLE) using GIS software and RS data. Remote Sensing data such as annual mean precipitation, land-use land-cover, and soil map, digital elevation model map were used to determine the USLE factor values. The average annual rainfall data was derived from the widely used climate dataset CRU TS (Climatic Research Unit gridded Time Series) and converted to rainfall erosivity factor. Soil Erodibility Factor Soil (K) was calculated from FAO soil data “Digital Soil Map of the World - ESRI shapefile format”. Topographic Factor (LS) was delineated from a 30m digital elevation model. Cover Factor (C) and Support Practice Factor (P) were estimated from a 20m Ethiopia Sentinel2 Land-use Land-cover year, 2016. The study classified the Guder watersheds into different kinds of severity classes for prioritization of soil and water management options and conservation strategy. The mean annual soil eroded for the whole sub-basin was estimated at 25.23 tha^−1^y^−1^. The study output outcomes demonstrated that about 0.1% (426ha) 6.9% (46764 ha), 8.9% (60055 ha), and 19.8 % (134320ha) have been under Catastrophic, very severe, severe, high erosion severity class respectively. About half of the Guder sub-basin has been underneath a very slight erosion. Nevertheless, the area covered by very severe erosion was 6.9%, and the annual percent of sum-total soil erosion accounted for was 46.86%. The second and third in magnitude soil lost annually from the sub-basin with regards to per cent of total soil loss were severe (26.53%), and high (21.53%) respectively. In only 7% of the area under investigation, soil erosion estimated was to go beyond 100 t/ha/yr. erosion rate. District wise erosion affected and hotspot areas were identified: Middle of Steep slopes Mountainous parts of Ginde Beret, Jeldu, Ifata, Ambo, parts Ababo and Horo Guduru located in the study area borderline, Toke Kutaye, along the boundary of Midakegn and Cheliya were found in severe to very severe erosion. Finally, the study proposed that the government, policymakers, and soil and water management agents plan and implement the conservation measures and give awareness to stakeholders for optimum use of limited precious resources.

## 1 Introduction

Soil loss creates a universal ecological and economic crisis (Allen et al., 1991; Pimentel et al., 1995; Bewket and Teferi, 2009; Tosic et al., 2011; Nadew et al., 2018; Jiu et al., 2019; Tessema et al., 2020). It results in the loss of fertile topsoil, lessens the productivity of the soil and thus causes a hazard to global food security (Jie et al., 2002; Pimentel, 2006; Daniel, 2015; Gupta, 2019). Likewise, it can affect harmfully the water storage structure volume of catchments, service year of manmade dams, reservoirs, quality of surface water resources, aesthetical beauty of the landscape and hydrological balance in general. As far as soil is a nonrenewable natural resource, soil erosion remains a critical environmental problem (Bewket and Teferi, 2009; Daniel, 2015; Gupta, 2019).

In the Upper Blue Nile River (UBNR) Basin of Ethiopia, soil loss by water is found to be a severe risk to the countrywide economy (Sonneveld, 2002; Havnevik et al., 2007; Bewket and Teferi, 2009; Worku and Tripathi, 2015). As stated by (Sonneveld, 2002), the estimated economic loss of soil erosion is around 1 billion US dollar per year; whereas the World Bank, 2007 state that the minimum annual cost of soil loss ranges between 2-3 % of the country's agricultural GDP. These studies visibly indicate the threat of food insecurity in the country as a result of soil erosion. The cause of the extreme rate of soil loss has been uncontrolled exploitation of land for activities like extensive removal of vegetation for fuel, and frequent expansion of farming and population growth rate (Tekle and Hedlund, 2000; Zeleke, 2000; Amsalu et al., 2007; Bewket and Teferi, 2009).

On the contrary, despite the substantial efforts done to develop and promote different kinds of soil and water conservation and management measures, acceptance, implementation and sustained use by the stakeholders have not been widespread for several reasons (Shiferaw and Holden, 1999; Bekele and Drake, 2003; A. Amsalu and de Graaff, 2007; Anley et al., 2007; Bewket and Teferi, 2009). However, natural resources conservation is a must for sustainable use, soil and water conservation actions have been not applied in this watershed, maybe due to negligence, lack of awareness, capital investment, and participatory approach in planning. So, government, policymakers, development agents, natural resource conservation agents, technicians, landowners or local farmers must integrate to plan for the successful implementation of natural resource management and conservation strategy.

Therefore, soil loss remains a problem to be confronted by the government's efforts to ensure food security, reduce poverty and ecological sustainability. Planning, implementation, and execution of effective soil and water conservation require a thorough understanding of the extent, risk and spatial distribution of the problem. It has significance to soil and water conservation agencies, government, and policymakers, develop-ment agents and field experts, and landowners for a targeted conservation involvement by identifying the most highly endangered landscapes for the setting of priorities. The RUSLE uses the same formula as the USLE, but with improvements in the estimation of many factors. RUSLE can take into account more complex combinations of tillage practices and cultivation practices as well as a wider variety of slope forms (Renard et al., 1994). According to the study conducted by different scholars, the estimation of soil erosion rate by USLE and RUSLE gives a similar erosion pattern with relatively similar results (Djoukbala et al., 2019; Evans, 2006; Mondal et al., 2018). The main objective of this research was to evaluate soil erosion hazards in a Guder watershed of the Blue Nile River (BNR) Basin using the simplest erosion evaluation model USLE, integrated with RS and GIS. USLE has been universally applied all over the continents (Hui et al., 2010; Zhang et al., 2011; Alewell et al., 2019) due to its simplicity and small input data requirement.

In this study, GIS and RS input data were applied to assess soil erosion severity class, ranking priority for treatment district-wise erosion-affected hotspot regions were identified for quick planning, and implementation of natural resource conservation practices and technology regardless of budget constraints. Finally, the study would give evidence for the government, policymakers, kebele administration office, development agents, and the agents responsible for soil erosion.

The final result of the investigation would benefit management policies to keep the soil from further destruction which is difficult for reclamation. It will also create soil and water conservation awareness in the society living within the border of the study area for the optimum use of soil for developmental purposes, and the sustainable usage of the available natural resource.

## 2 Materials and methods

### 2.1 Location of the Guder sub-basin

**Figure 1:**
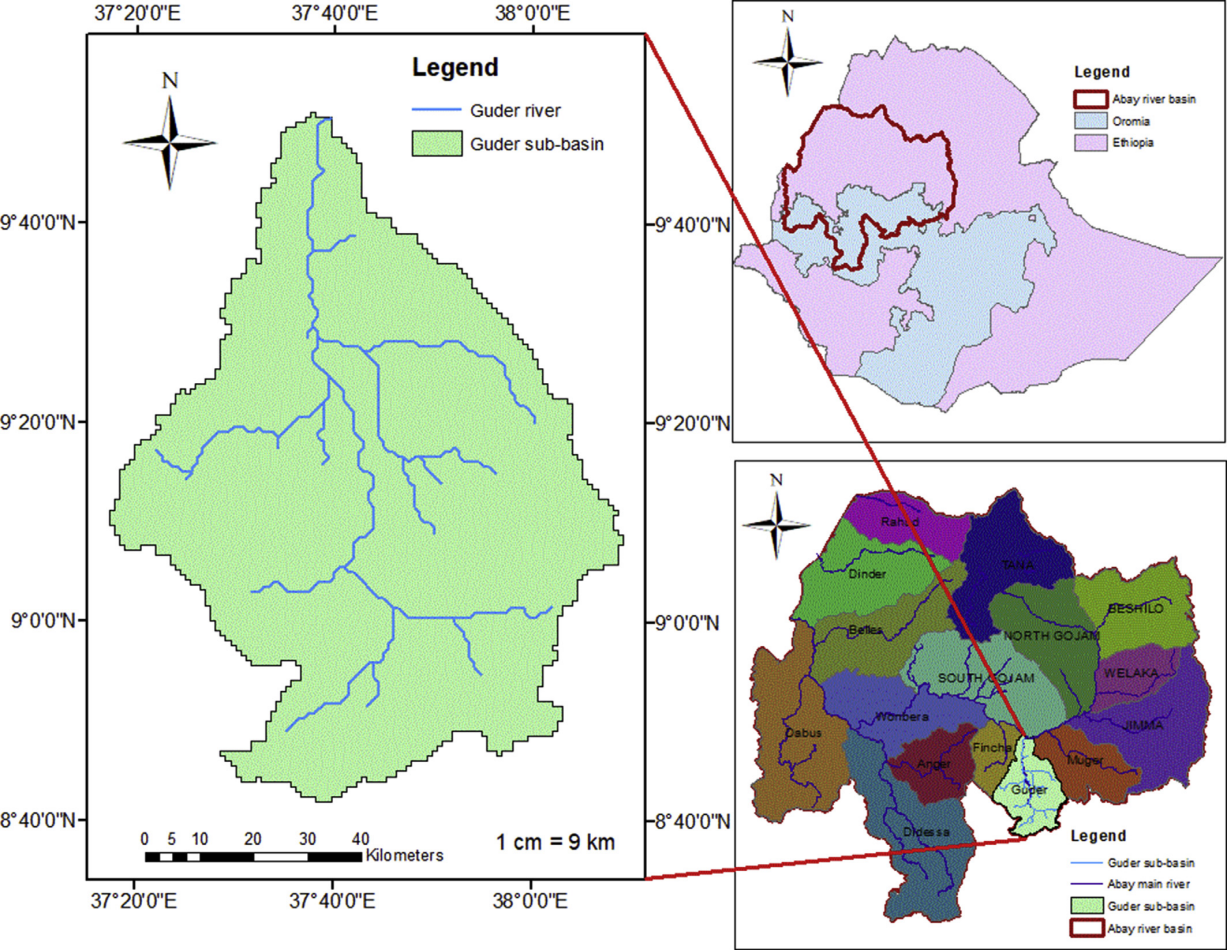
Geographical Location of the study area.

This watershed is geographically found in 8° 40′ 00″ to 9° 52′ 00″ N latitude and 37° 15′ 00″to 38° 10′ 00″ E longitude (Figure 1); and it is among one of the tributaries of the Abbay River basin of Ethiopia (Duguma et al., 2020) or UBN River. The Guder sub-basin shares borderlines with the Muger sub-basin to the eastward, the Awash Basin to the southward direction, and the Fincha River sub-basin to the westward direction. The Guder sub-basin has a drainage basin area extent of about 6785 square kilometers (Fentaw, 2018; Nadew’ et al., 2019; Subash et al., 2017). The climate of the Guder sub-basin is distinguished by unimodal with one rainy and one dry season. The rainy season extends from May to October and the dry season from November to April. The high rainfall intensity and, or amount obtained in July and August (Muleta and Biru, 2019b; Nadew’ et al., 2019); and the mean annual temperature received by the Guder watershed range between 6.5 °C and 30 °C.

**Figure 2:**
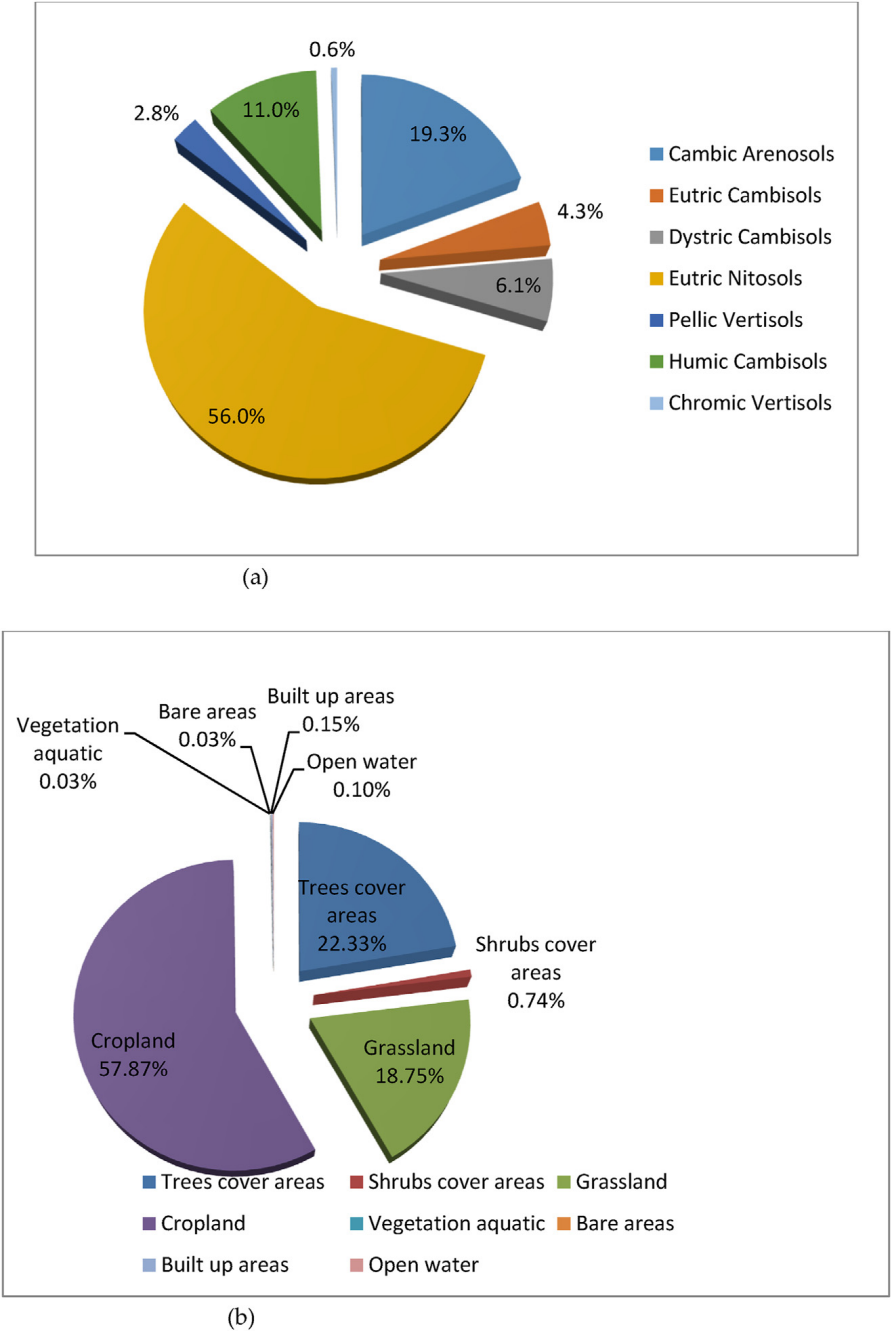
(a) The soil types of the Guder sub-basin according to the harmonized FAO world soil classification and (b) Land use/cover of the study area.

The Guder sub-basin drains into the Upper Blue Nile River (UBNR) Basin, and the Blue Nile River is the major and very important river in Ethiopia by volume and size of the river (Muleta and Biru, 2019a, Muleta and Biru, 2019b). The dominant soil types of Guder watersheds are Eutric Nitosols (56%), followed by Cambic Arenosols (19.3%), Humic Cambisols (11.0%), Dystric Cambisols (6.1%), Eutric Cambisols (4.3%) according to FAO soil classifications (Figure 2(a)). The main and dominant economic activities for the livelihood of the people are agriculture involving crop and livestock production which is mainly subsistent, petty trade and forest product collection and sale are some off-farm activities. According to Sentinel-2 land-use land-cover 2016, the major land use/cover of the Guder sub-basin is dominated by cropland/agriculture and covers (57.87%) of the whole watersheds; while Trees cover areas is (22.33%), and Grassland covers (18.75%) portion of the area respectively (Figure 2(b)). The average altitude of the area varies from 1144 to 3288 m above sea level as delineated by a 30m spatial resolution and it receives an annual mean rainfall of between 812.0mm to 1699.0mm (Duguma and Duguma, 2022). The Expansion of irrigation activities has been extremely practiced in the Guder watersheds (Subash et al., 2017).

The whole population of the sub-basin is about 130,500 of which the numbers male and female are 64,881 and 65,619 respectively. The main economic activity in the sub-basin is crop production or agriculture and livestock production (Duguma and Duguma, 2022; Muleta and Biru, 2019b; Subash, 2017). As stated by Muleta and Biru (2019a), landscape-level watershed conservation was recommended to enhance ecosystem services. As there is soil quality deterioration in the study area, the rural areas are recommended to protect the soil erosion by constructing different soil and water conservation measures (Subash, 2017). As stated by World Bank (2007) and other scholars attention must be given to protecting the soil erosion as it results in economic and social hazards to the people living in the watersheds and the country in general. So, if the soil has no protection measures it results in the threat of food insecurity in the country as a result of soil erosion.

As soil loss is one of the global environmental hazards which limits human existence and has a negative consequence on worldwide socio-economic sustainable development. Soil erosion destroys land resources, causes pollution, disturbs the utilization and recycling of water resources, deteriorates the environment, and results in natural disasters (Sun et al., 2020). Hence soil Erosion modelling has been important for sustainable environmental management (Majoro et al., 2020).

### 2.2 Soil erosion modelling

The USLE method of erosion modelling was developed and applied to approximate the mean annual soil eroded from Guder watersheds of the Upper Blue Nile River (UBNR) Basin. About five (5) GIS and RS data such as Rainfall erosivity (R) Factor, Soil Erodibility Factor Soil (K), Topographic Factors (LS), Cover Factor (C) Supporting Practice Factor (P) were applied to run the USLE model. USLE equation is expressed as follows:

(1) A=R∗K∗LS∗C∗P

**Figure 3:**
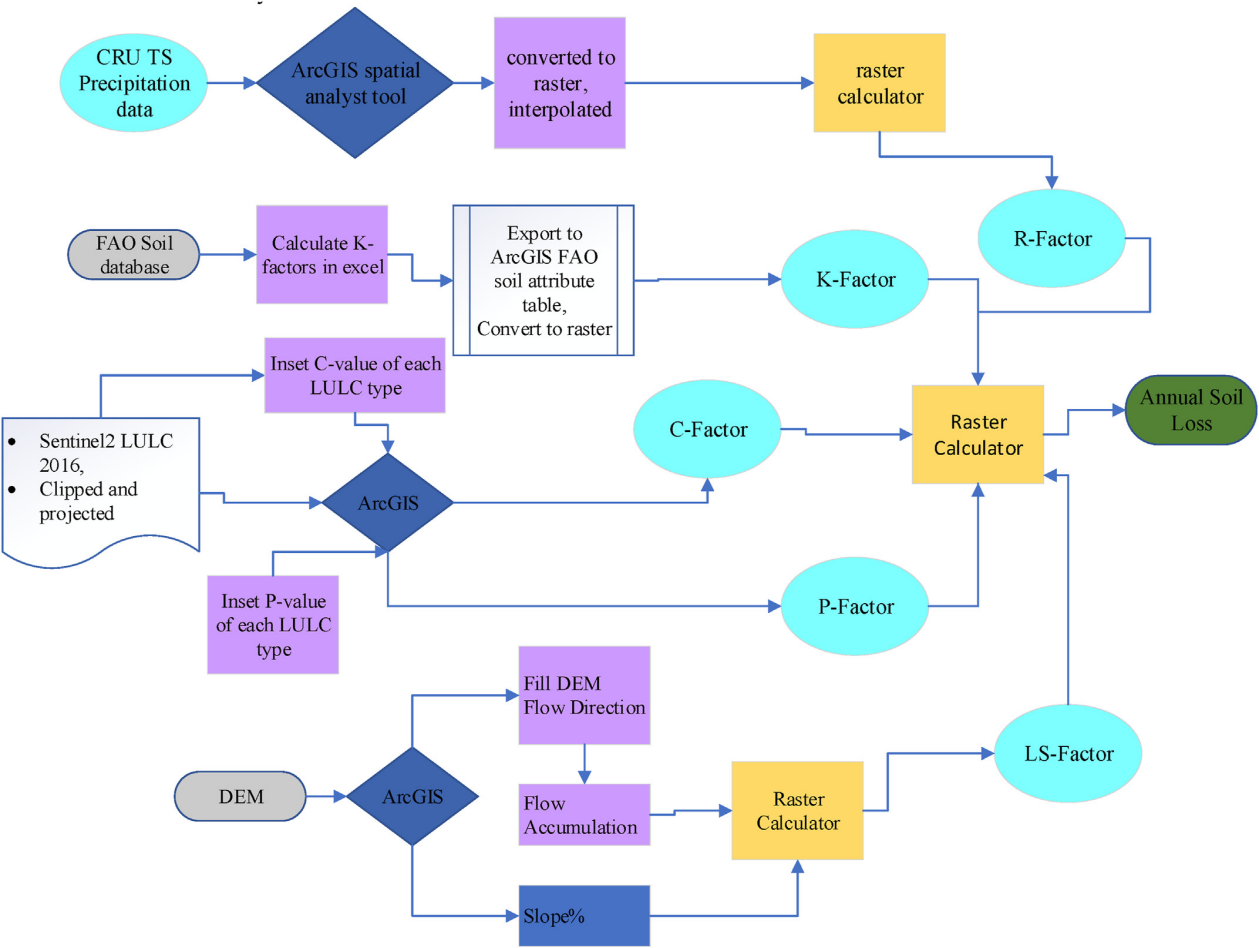
Work flow diagram.

Where, A is annual soil loss (t·ha^−1^yr.^−1^); R is the rainfall erosivity factor (MJ·mm ha^−1^h^−1^); K is the soil erodibility factor (t·h·MJ^−1^mm^−1^); LS is the slope factor; C is the soil management factor; P is the supporting practices factor. The workflow diagram which shows the step by step of how it has arrived at the result was briefly indicated in (Figure 3) below. Finally, the annual soil loss was reclassified in the ArcGIS of spatial analyst tool to describe the results more precisely.

#### 2.2.1 Rainfall erosivity (R) factor

The R factor denotes the rainfall and runoff's impact on soil (Hui et al., 2010; Lee, 2004; Onori et al., 2006; Prasannakumar et al., 2011; Talchabhadel et al., 2020) that is the direct product of rainfall/storm energy (E) and the maximum 30-minute intensity (I_30_). For areas that have no detailed climate data, R could be calculated using different empirical equations as there is no rainfall intensity for the study area. So, an empirical equation with the relation between mean annual rainfall and R-values developed by (Grunder, 1988; Hurni, 1988), which is easily available was used to determine the R-value. This equation of rainfall erosivity factor was applied and implanted in different watersheds of Ethiopia (Ali and Hagos, 2016; Bewket and Teferi, 2009; Kebede et al., 2021; Mohammed and Asmamaw, 2021; Tiruneh and Ayalew, 2016). The relationship was given by the following equation:

(2) R=−8.12+0.562∗P

Where, R is the rainfall erosivity factor, whereas P is the average annual rainfall.

**Figure 4:**
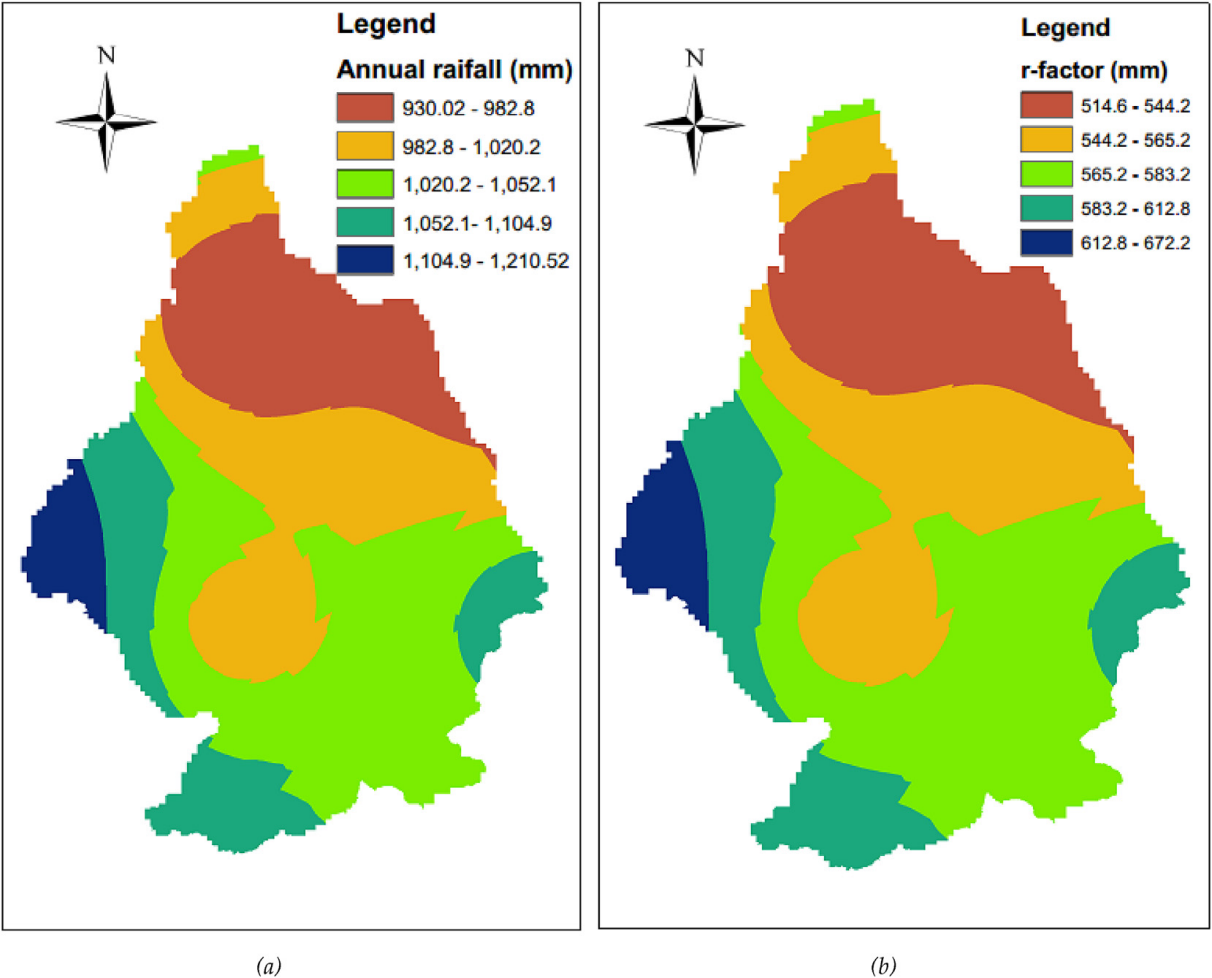
*(a) Mean annual rainfall and (b) rainfall erosivity factor*.

As the rain gauge measured rainfall data were rarely found in the watershed, remote sensing data was applied. The widely used climate dataset CRU TS (Climatic Research Unit gridded Time Series) of spatial resolution 0.5° latitude by 0.5° longitude monthly high-resolution gridded multivariate climate dataset Version 4.05 was accessed and downloaded on the website https://crudata.uea.ac.uk/cru/data/hrg/cru_ts_4.05/on March 28, 2022, in NetCDF format. This monthly rainfall in the NetCDF file was converted in raster, composite, and interpolated in ArcGIS spatial analyst tool. The Inverse Distance Weighted (IDW) interpolation technique was applied as it is easy to define and therefore easy to understand the results. It was converted to r-factor in the raster calculator of map algebra in the spatial analyst tool. The 10-year (2011–2020) total of rainfall was calculated in cell statistics under the spatial analyst tool. Note that the mean annual rainfall was calculated in the raster calculator of the spatial analyst tool. The final delineated rainfall erosivity (R) factor ready for the USLE model was indicated in Figure 4(b) below.

**Figure 5:**
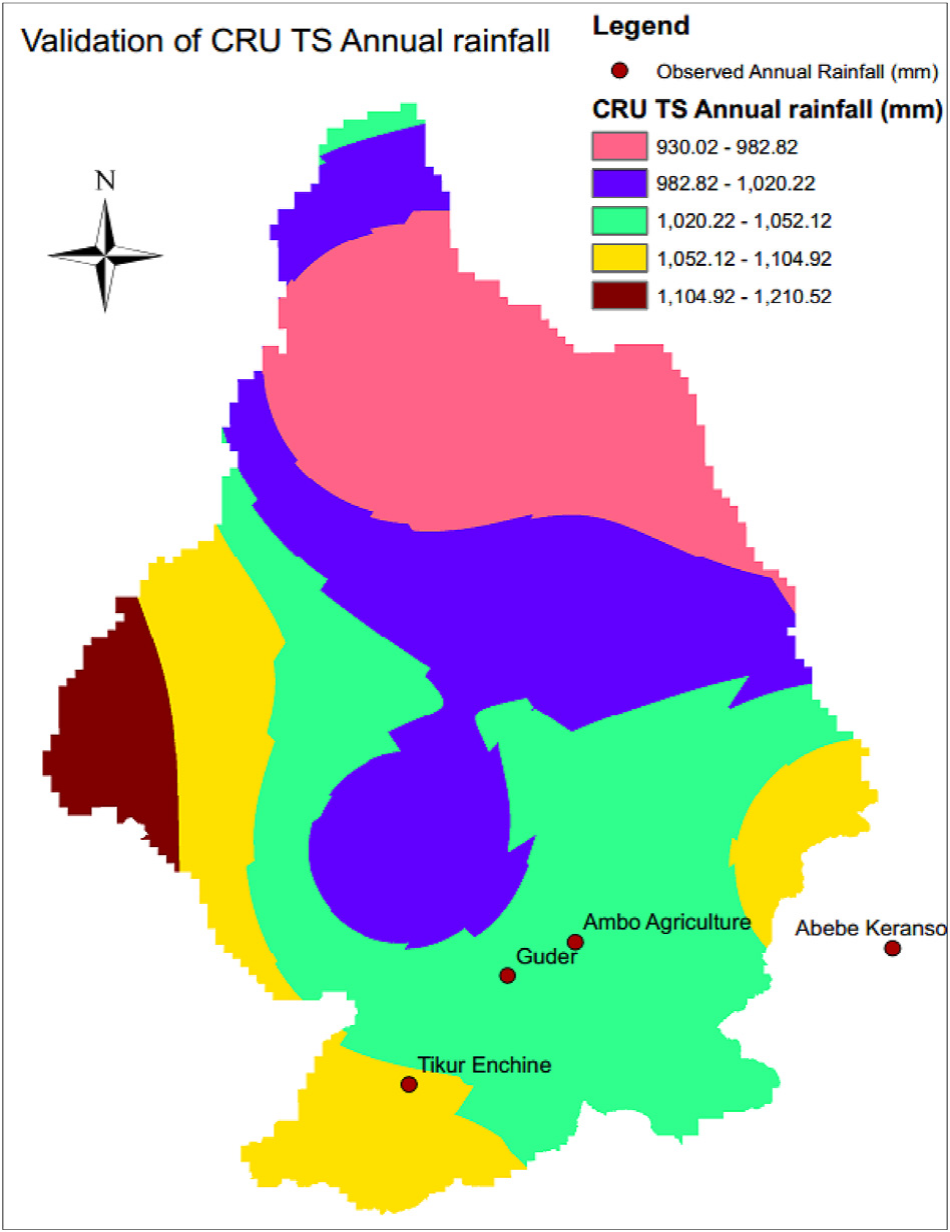
Location of observed rainfall.

This CRU TS (Climatic Research Unit gridded Time Series) dataset has been applied in the sab-basin of Ethiopia by different scholars for example (Asfaw et al., 2018). The study collected observed rainfall and converted it to average annual rainfall. Besides, the study tried to find the location of the observed gauging station and matched it with the corresponding CRU TS value in ArcGIS. The Observed data and the corresponding CRU TS data were tabulated in Figure 5 , Table 1 below.

**Table 1:** Observed vs CRU TS data of four stations.

SN.	Site_Name	longitude (deg.)	latitude (deg.)	Elevation(m)	Observed Rainfall (mm)	CRU TS Rainfall (mm)
1	Tikur Enchine	37.668	8.836	2467	1106.0	1051.6
2	Abebe Keranso	38.169	8.978	2456	1119.2	1080.8
3	Ambo Agriculture	37.840	8.985	2068	1080.3	1029.6
4	Guder	37.770	8.950	2040	1098.2	1033.2

**Figure 6:**
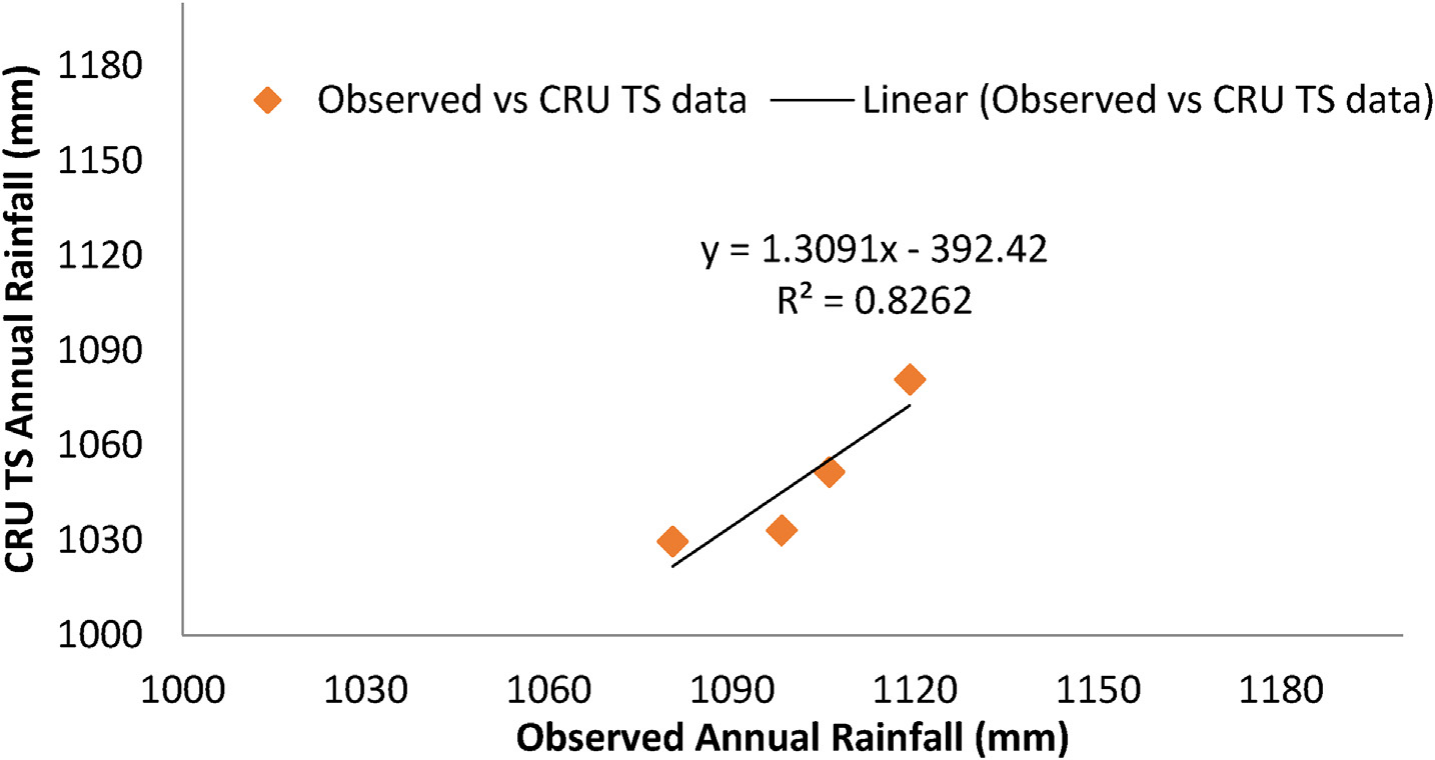
Relationship between observed and CRU TS rainfall.

CRU TS dataset was validated by the standard procedure for example the Pearson correlation coefficient (r), bias, Mean Absolute Error (MAE) and the Root-Mean-Square Error (RMSE), and Efficiency (Eff) (Ageet et al., 2022; Atiah et al., 2020; Ayehu et al., 2018; Dinku et al., 2018). About four gauging stations are available to validate this satellite data. These stations by name are Tikur Enchine, Abebe Keranso, Ambo Agriculture, and Guder. This study validated the CRU TS using correlation coefficient (r^2^) (Figure 6), MAE, RMSE, Eff and bias of 0.83, -13.03, 4.81, 0.037, and 0.95 respectively. Thus the study was validated by statistically accepted values as indicated by (Ageet et al., 2022; Atiah et al., 2020; Ayehu et al., 2018; Dinku et al., 2018).

#### 2.2.2 Soil erodibility factor

Soil erodibility relies on soil and/or geological characteristics, such as parent material, soil texture, structure or porosity of soil surface horizons and per cent of organic matter content (Wang et al., 2001; Foltz et al., 2011). Soil Erodibility Factor (K) values were calculated from the Food and Agriculture Organization (FAO) of the United Nations soil data “Digital Soil Map of the World - ESRI shapefile format”. It was get accessed on February 20, 2022, and downloaded from the website: https://storage.googleapis.com/fao-maps-catalog-data/uuid/446ed430-8383-11db-b9b2-000d939bc5d8/resources/DSMW.zip in zip format. The soil map of the Guder sub-basin was clipped and projected from Worldwide ArcGIS soil data. The k-value parameter and sub-parameters (Eqs. (3), (4), (5), and (6)) were calculated in Microsoft Excel and imported to the study soil map attribute table in ArcGIS 10.1. The K-Value formula given by (Neitsch et al., 2005) was used in this study to calculate k-factor. It is given by:

(3) $$K_{USLE}=f_{csand}*f_{cl-si}*f_{org}*f_{hisand}$$

where: $f_{csand}$ is a factor, which lessens the K factor in soils together with high coarse-sand content, greater for soils with smaller or limited sand; $f_{cl-si}\;$ provides the lowest K-value for soils with the highest clay-to-silt percent ratios; $f_{org}$ lowers K-values in soils with greater organic carbon content, whereas $f_{hisand}$ diminishes K-values for soils with the greatest degree of sand content:

(4) $$f_{csand}=\left\{0.2+0.3*exp\left[-0.256.m_{s}*\left(1-\frac{m_{silt}}{100}\right)\right]\right\}$$

$$f_{cl-si}={\left(\frac{m_{silt}}{m_{c}+m_{silt}}\right)}^{0.3}$$

(5) $$f_{org}=\left(1-\frac{0.25*orgC}{orgC+exp\left[3.72-2.95*orgC\right]}\right)\;$$

(6) $$f_{hisand}=1-\frac{0.7*\left(1-\frac{m_{s}}{100}\right)}{\left(1-\frac{m_{s}}{100}\right)+exp\left[-5.51+22.9*\left(1-\frac{m_{s}}{100}\right)\right]}$$

where: $m_{s}$ is the sand percentage content (0.05–2.00 mm diameter) [%];

✓ $m_{silt}$ is the silt percentage content (0.002–0.05 mm diameter) [%];
✓ $m_{c}$ is the clay percentage content (<0.002 mm diameter) [%];
✓ orgC is the organic carbon percentage (SOC) content [%].

**Figure 7:**
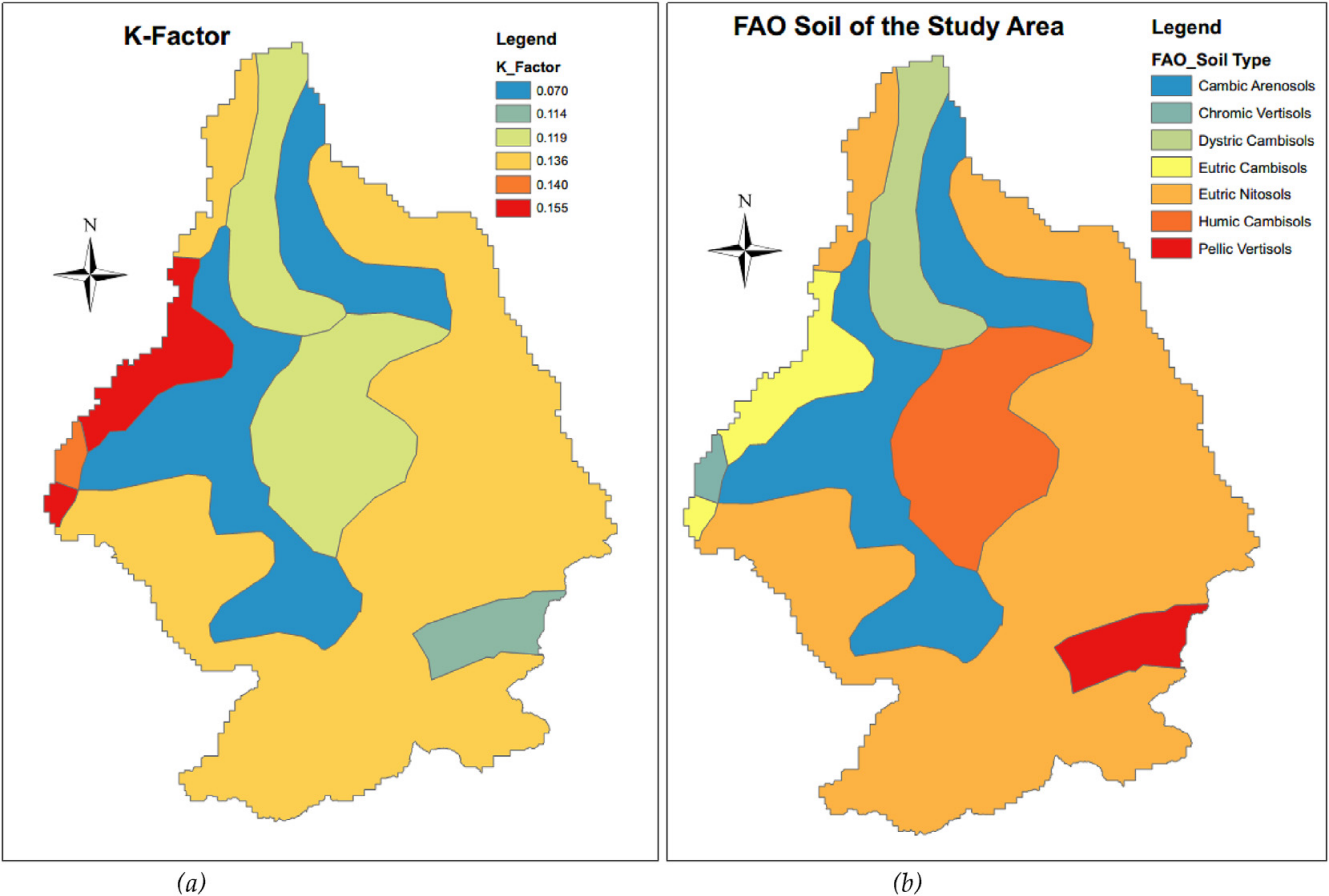
(a) Soil Erodibility Factor (b) (FAO) soil data.

The prepared soil erodibility factor Soil (K) map was shown in Figure 7(a) below and Figure 7(b) is the FAO soil type map of the study area.

#### 2.2.3 Cover factor (C)

**Figure 8:**
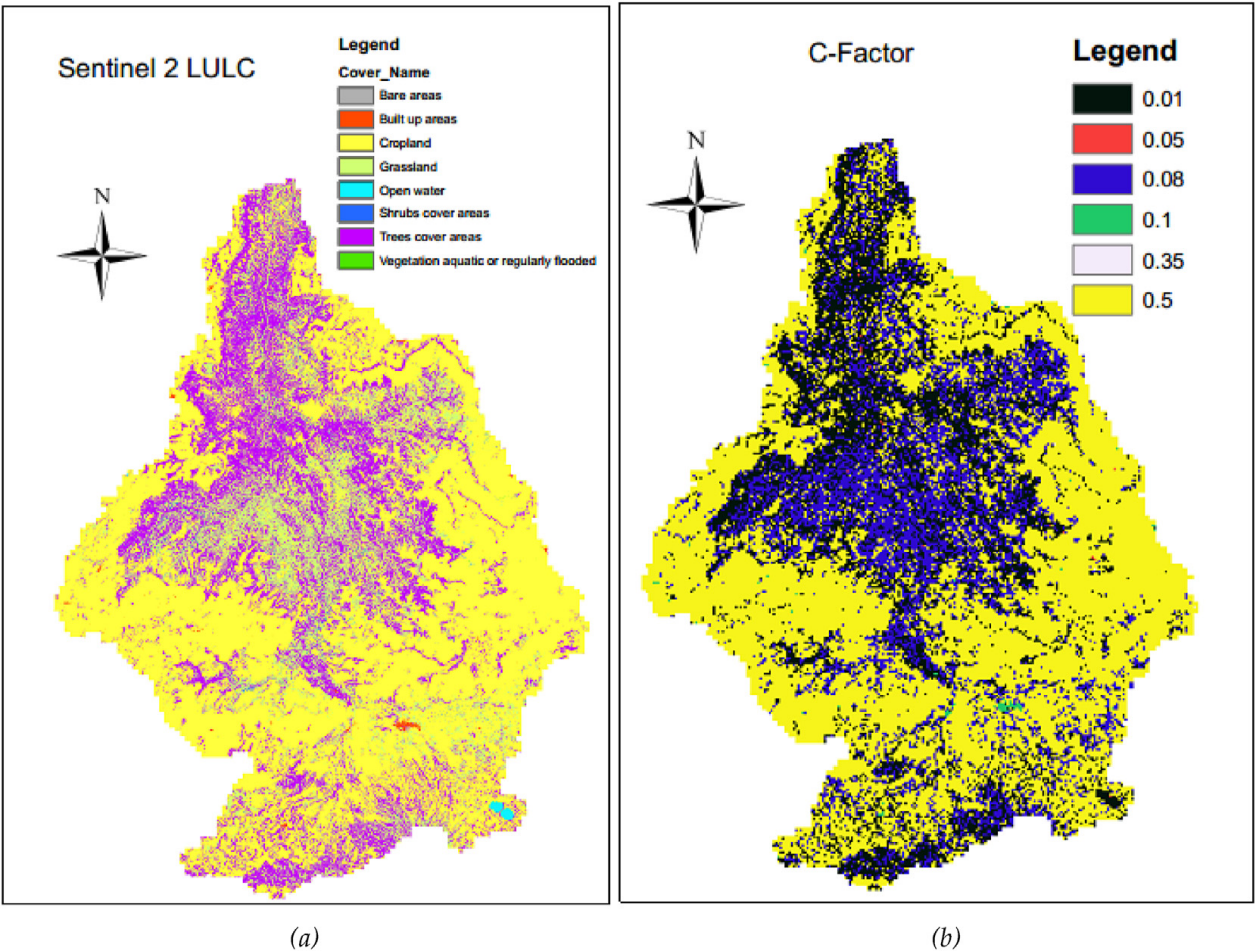
(a) Ethiopia_sentinel2_lulc 2016 (b) Cover Factor (C).

The C factor in the USLE represents the effect of cropping and management practices in agricultural management and the effect of the ground, tree, and grass covers in decreasing soil erosion in nonagricultural situations (Wang et al., 2002). It quantifies the collective impact of the whole interconnected cover and crop management variables (Folly et al., 1996). The USLE Cover factor is defined as the relative impact of vegetation reducing soil erosion rate in agricultural management, and it is the impact of the ground surface, tree, and grass covers in decreasing soil erosion in non-agricultural conditions (Anache et al., 2014; Kuo et al., 2016; Wang et al., 2002). The Land-use land-cover spatial resolution 20m by 20m of ‘Ethiopia Sentinel-2 Land-use and land-cover 2016’ got accessed and downloaded on March 20, 2021, from http://geoportal.rcmrd.org/layers/servir%3Aethiopia_sentinel2_lulc2016. The land-use land cover of the Guder watershed was clipped and projected in ArcGIS 10.1 version. About eight land-use and land-cover classes were recognized from the clipped land-use and land-cover of the study area. The cover factor and support practice factor figure of each thematic sub-factor is estimated from previously published articles and assigned accordingly. The C-value map of each land cover type is assigned as found in Table 2 and Figure 8 below.

**Table 2:** P-Factor and C-Factor values different sources.

SN.	Land cover type	C-value	Source	P-value	Source
1	Trees cover areas	0.01	(Bakker et al., 2008; Teng et al., 2016; Tessema et al., 2020; Yang et al., 2003)	1.0	(Brema and Hauzinger, 2016; Chen et al., 2019; Jiu et al., 2019; Molla and Sisheber, 2017)
2	Shrubs cover areas	0.01	(Bakker et al., 2008; Gelagay and Minale, 2016; Teng et al., 2016)	1.0	(Molla and Sisheber, 2017; Chen et al., 2019; Jiu et al., 2019)
3	Grassland	0.08	(Yang et al., 2003; Theobald et al., 2010; Chuenchum et al., 2020)	1.0	(Chen et al., 2019; Jiu et al., 2019)
4	Cropland	0.5	(Yang et al., 2003; Theobald et al., 2010)	0.8	(Yang et al., 2003; Haregeweyn et al., 2017; Chuenchum et al., 2020; López-García et al., 2020)
5	Vegetation aquatic or regularly flooded	0.05	(Yang et al., 2003; Teng et al., 2016; Chuenchum et al., 2020)	1.0	(Yang et al., 2003; Haregeweyn et al., 2017; Chen et al., 2019; Chuenchum et al., 2020)
6	Bare areas	0.35	(Yang et al., 2003; Teng et al., 2016; Altü rk et al., 2016; Chuenchum et al., 2020)	1.0	(Yang et al., 2003; Naqvi et al., 2013; Haregeweyn et al., 2017; Chen et al., 2019; Chuenchum et al., 2020)
7	Built up areas	0.1	(Yang et al., 2003; Bakker et al., 2008; Gemechu, 2016)	1.0	(Naqvi et al., 2013; Brema and Hauzinger, 2016)
8	Open water	0.01	(Yang et al., 2003; Gwapedza et al., 2018; Chuenchum et al., 2020)	1.0	(Yang et al., 2003; Naqvi et al., 2013; Haregeweyn et al., 2017; Chuenchum et al., 2020)

^1^The C-Factor and P-Factors searched, and reviewed different scientific manuscripts and cited and used accordingly. The values used by most scholars were taken, while few researchers used a different value that may be a slight difference from the stated values.

#### 2.2.4 Supporting practice factor (P)

**Figure 9:**
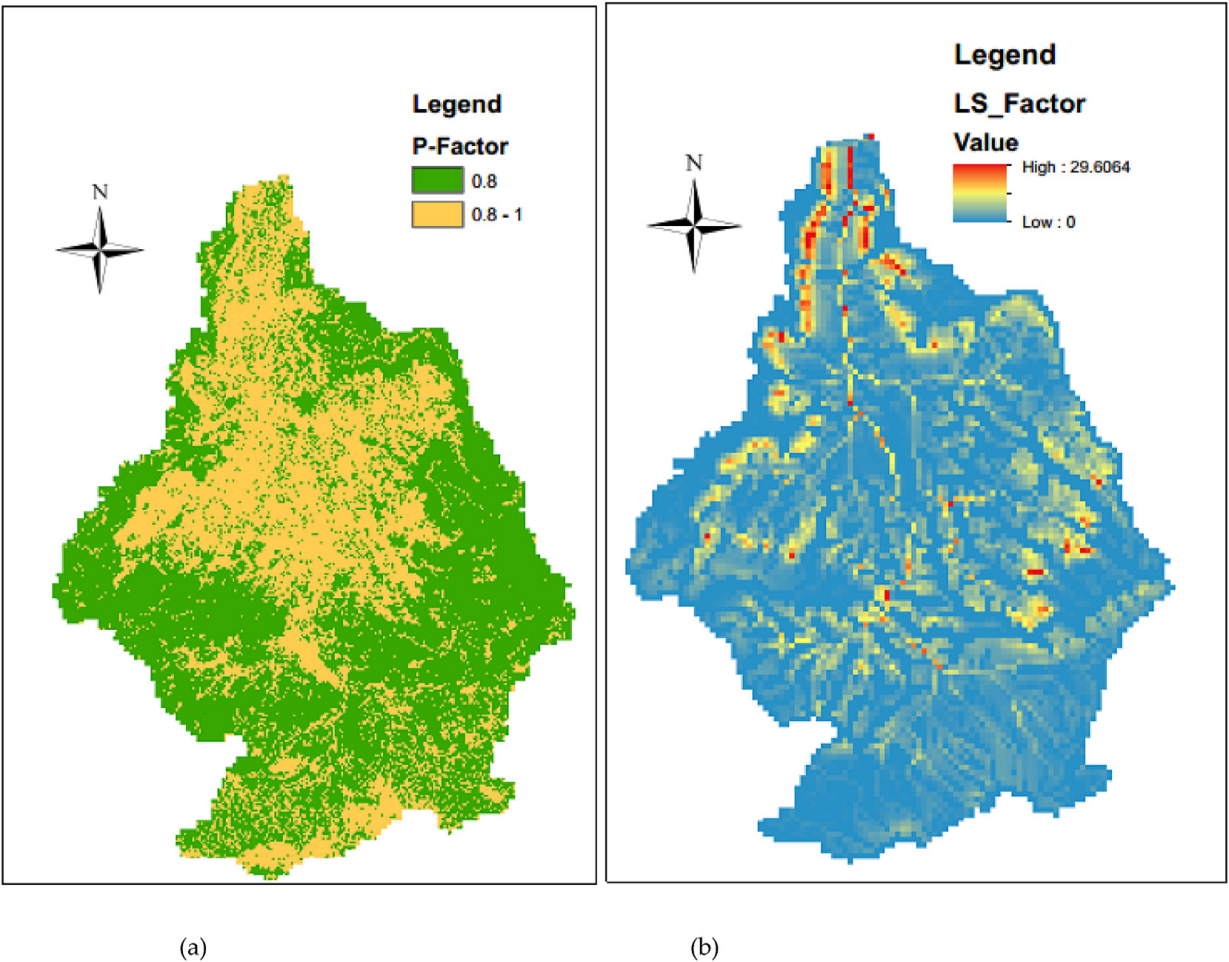
(a) Supporting practice factor (P) (b) topographic factors (LS).

The supporting practice factor is the percentage ratio of correspondent soil loss to specified farming practice, through the upsloping surface and downsloping tillages altering the flow direction, pattern, and volume of surface runoff (Morgan, 2009). In the lack of permanent soil and water management practicabilities data, the (P) value has been derived from either land-use landcover or slope; or a combination of them (Bewket and Teferi, 2009; Wischmeier and Smith, 1978). Therefore, the values were designated to each soil management practice and corresponding values as recommended by numerous researchers worldwide (Table 2) above and the corresponding Supporting Practice Factor (P) map was indicated in Figure 9(a).

#### 2.2.5 Topographic Factors (LS)

The ASTER DEM retrieved from (http://www.gdem.aster.ersdac.or.jp/search.jsp) was downloaded, clipped and projected to the study are-a. The data was acquired on March 08, 2016. The LS-factor was calculated from the DEM as the procedure is indicated in Figure 3.

The effect of both slope steepness and length on the quantity of the erosion is together known as the topographic factor (LS) (Kinnell, 2010; Du et al., 2015). The appropriate value can be estimated on nomographs (Wischmeier and Smith, 1978; Morgan, 2009) or the equation:

(7) $$LS={\left(\frac{x}{22.13}\right)}^{0.4}*\left(0.065+0.045s+0.0065s^{2}\right)$$

where, LS is the slope factor, x is the slope length (m) and s is the slope angle in per cent (Bewket and Teferi, 2009; Morgan, 2009). Therefore, a DEM-based procedure developed was applied to resolve the difficulties arising from the estimation of the LS-factor on a regional scale. The algorithms of the steps can apply the use of the raster grid flow accumulation and slope in per cent. The equation is modified as it can be processed in ArcMap as follows:

(8) LS=Power(FA∗cellsize/22.13,0.4)∗(0.065+0.045∗Slope%+0.0065∗Slope%∗Slope%)

Where, FA is flow-accumulation, Slope% is the slope inclination angle in per cent and the cell size of this study was 30m and x in Eq. (7) is the product of flow accumulation and cell size (T. Amsalu and Mengaw, 2014; Teng et al., 2016; Tessema et al., 2020; Tosic et al., 2011).

## 3 Results and discussion

### 3.1 Soil loss potential map/soil erosion hazard mapping

**Figure 10:**
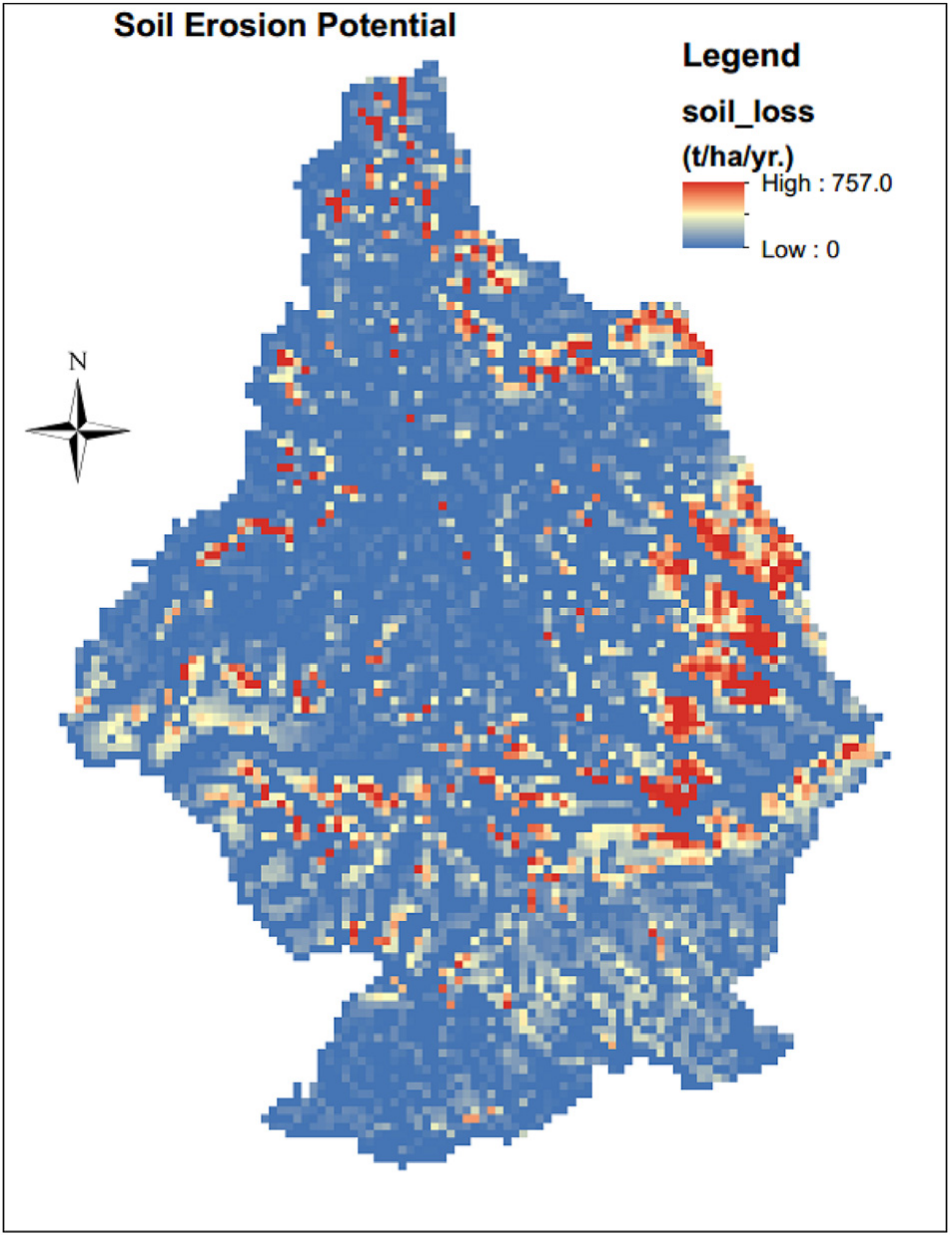
Actual estimated soil loss.

**Figure 11:**
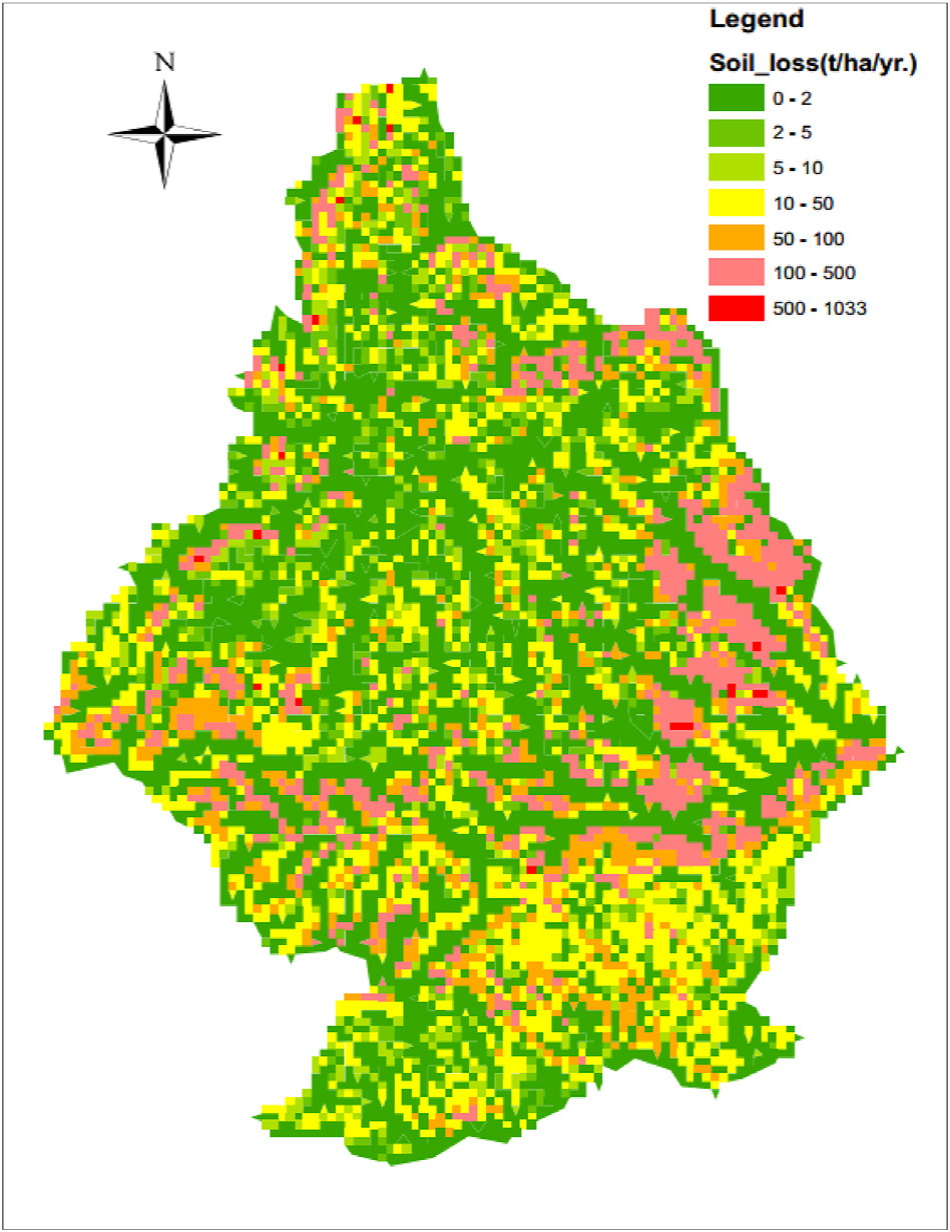
Classified soil erosion rate map.

The annual soil loss magnitude was determined by multiplying the individual USLE factor in the in-raster calculator part of ArcGIS10.1, a spatial analyst tool using Eq. (1), resulting in the erosion potential map shown in (Figure 10). The estimated annual soil loss values ranged from 0 to 757 t^−1^ha^−1^y^−1^. The highest values were at the periphery of the Guder watersheds and the basin was categorized into seven classes to identify the erosion hotspot areas (Figure 11). The mean annual soil eroded for the whole sub-basin was estimated at 25.23 tha^−1^y^−1^ and about 16.3 million tons of soil may be eroded from the basin annually. About 58.2% of the Guder sub-basin is found between very slight to slight erosion, moderate (6.1%), high (19.8%), Severe (8.9%), and Very severe (6.9%) severity classes according to (Morgan, 2009) erosion classification. Nearly half (58.2%) of the study area is under a very slight to slight erosion rate Table 3 and Figure 11.

**Table 3:** Soil erosion severity according to (Morgan, 2009).

Code	Soil loss (t ha^−1^yr^−1^)	Code Class	Area Covered (ha)	Per cent of total area
1	<2	Very slight	352465	51.9%
2	2–5	slight	42962	6.3%
3	5–10	Moderate	41509	6.1%
4	10–50	high	134320	19.8%
5	50–100	Severe	60055	8.9%
6	100–500	Very severe	46764	6.9%
7	>500	Catastrophic	426	0.1%

Classification in ArcGIS was based on the natural breaks type of classification.

Based on the estimated annual soil erosion rates, the sub-basin was categorized into seven erosion severity classes (Table 4). Thus, 15.8% area of the sub-basin was estimated to be affected by a severe to very severe erosion hazard, which ranges from 50 to 500 t/ha/yr. and catastrophic erosion class covers only 0.3% of the study area. High to Moderate severity class covers about a quarter of (25.9%) of the Guder sub-basin. And about half of the study area (58.2%) was found in the slight to very slight severity class. Severe or very severe, catastrophic erosion was observed in the margin and upstream segments of the watersheds because of the existence of escapements, hills & Steep Side slopes, slopes, and rock surfaces. On the other hand, overgrazing, and localized gullies formation, remain the dominant processes in utmost segments of the sub-basin (Subash et al., 2017).

**Table 4:** Priority, and severity classification for amendment of eroded areas.

Soil loss (t ha-1yr-1)	Priority classes	severity class	Area Covered (ha)	Per cent of the total area	Per cent of total soil loss
>500	I.	Catastrophic	426	0.1%	1.65%
100–500	II.	Very severe	46764	6.9%	46.86%
50–100	III.	Severe	60055	8.9%	26.53%
10–50	IV.	high	134320	19.8%	21.53%
5–10	V.	Moderate	41509	6.1%	1.98%
2–5	VI.	slight	42962	6.3%	1.01%
<2	VII.	Very slight	352465	51.9%	0.44%

As the socio-economic impact of this erosion is very high, the government, policymakers, and natural resource conservation agents must give attention to this study area. Currently, more than 130,500 people have been living within the boundary of the study area. If the sub-basin cannot be treated with different erosion control measures, sustainable production and productivity will be in question. The economic activity of the population living in the study area has been agriculture and livestock production. Both agriculture and livestock production depends on this precious natural resource. More than 80% Ethiopian population are also farmers and farm activity depends on soil. Unless different protection measure is applied, this erosion can harmfully affect the population within the sub-basin besides climate change. So, the sub-basin must be classified into different classes in order to make it suitable for applying different treatment measures.

### 3.2 Watershed treatment prioritization

**Figure 12:**
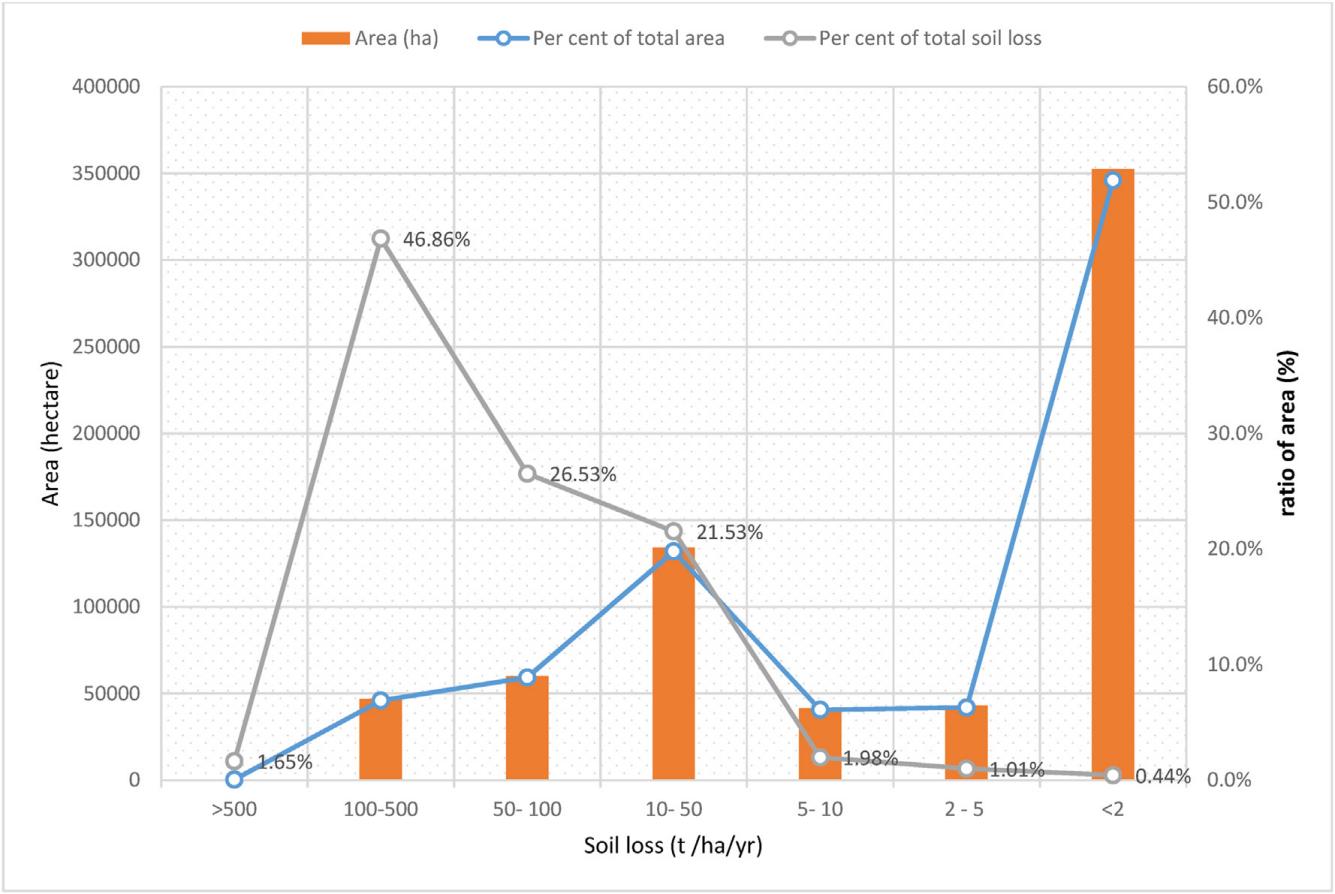
The graph of Soil erosion severity according to (Morgan, 2009), the per cent share of each class, and Per cent of total soil loss.

The soil erosion potential or hazard map Figure 12 and Table 4 clearly show that approximately the 41.8% sub-basin needs implementation of different forms of erosion control and intervention measures for sustainability of land use. Even though it requires resource considerations issue for participatory basin development, however, the study tried to show the priority to identify which erosion severity class need immediate attention for the applicability of soil conservation technologies. Despite the fact that resource constraints are a well-known problem in Ethiopia, the study identifies erosion hotspots to reduce the soil erosion rate and to give necessary information for government, policymakers, natural resource conservation agencies, development agents and stakeholders to give priorities to highly eroded areas. If control measures will be planned at erosion hotspot areas, erosion and sedimentation can be reduced significantly. Similarly, as can be observed from (Table 4) below, about 46.86%of total soil loss is from very severe erosion classification and 26.53% was from the Severe erosion class. So, if erosion control strategies are applied for a very severe erosion severity class, we can reduce the erosion rate significantly from the basin as about nearly half of the sub-basin is found in this erosion severity class.

Hence, it is essential to prioritize the watersheds for treatment with suitable soil and water erosion control measures. Furthermore, prioritization of watersheds means ranking it for the different watersheds as necessary to initiate soil and water conservation treatment option depending on the magnitude of mean annual soil loss.

The priority classes were assigned depending on the amount of soil lost per hectare; nevertheless, the percent of grand-total soil erosion from the sub-basin may be small. So, the primary and secondary priority classes were catastrophic and very severe for planning and implementation of the soil and water conservation actions. More importantly, as the consequence of drought expansion has been repeatedly observed in the country, especially, in these two years (2020–2022) thus government must apply soil, and water conservation policy besides green policy which is under implementation. In addition, since the soil is a nonrenewable natural resource, highly erodible areas must be given priority, because as the fertile topsoil is removed it may become difficult for afforestation amendment and result in the condition of bare land.

### 3.3 District/woreda and kebele-based classification

**Figure 13:**
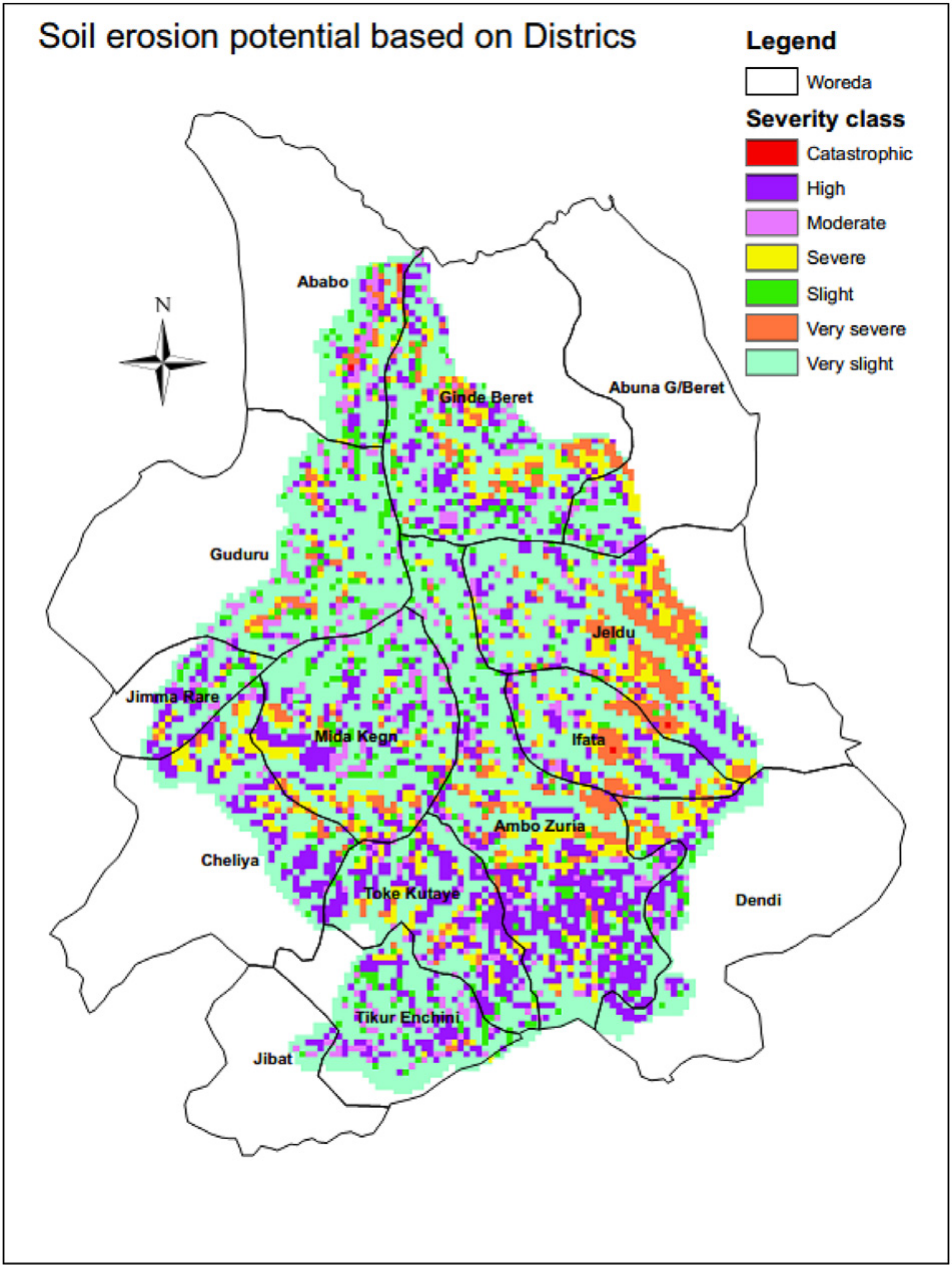
District-based Soil erosion potential or risk classification.

Guder watershed shares areas with about fourteen Woredas of Western Oromia. The study tried to identify the Woreda or kebele found from severe, very severe and catastrophic erosion for quick amendment of the affected Woreda or kebele. Steep Side slopes Mountainous parts of Ginde Beret (Kebele: Erjajo,Goro Jaleti&Abasebet,North Lega Mecha& Kiltu sembeta, Seke Yadi, Goro Mene Ega,damota, east Kere Sole, Haro, middle Dire Faji&Gemeda&Beke Bela, south Oula Agedadi, south Kere senkore), Middle of Jeldu Woreda (Kebele: Teso,Alike, Shukute, Chenchek Kebenaa,Gora Lelisa,Mek saleku,near boundary of Korchak Otcha,Korchachobi,Chabi Town,Direkebena, Herokakeli,Tulugurji, Odobad-esa,Herodegedaba), Ifata Woreda (Kebele: Amibal Tagodeti, boundary of Beke town &Somibo Chitu&Gicho&Haro Tuffiticha,Fulicha, Gute Sado), Ababo Guduru (Kebele: Chala Foka, Bikiltu Emibabo), and Horo Guduru (Kebele: at middle of Ref-Gudene,Ni-Barie-Ufe,West of Ref Toko Tane&Kenate Dinisa&Elamu Tareko), Ambo (Kebele: Dobi, Abe Doyo, Dagale Gatira, Golfofa, Tule, Gamosa Sato, Dek, Tero, Gelan wadesa), near boundary of Mida Kegn (Kebele: Ofenjo Toke laga, lanto Jareso, Aretato Kelega,Halelu Gosu) and Cheliya (Kebele: Wello Tamebara, Ole Sere, Gudata Gelalega, Dhangego) found from severe to catastrophic erosion severity class (Figure 13).

So, the government, policymakers, and natural resource conservation agents must give priority and attention to these areas (kebeles). Because this study shows that the areas were under severe to catastrophic erosion severity class. Unless remedial measures are not applied soon, they may go beyond treatment difficult. Though most of the study areas require soil and water conservation management technologies, priority must be given to highly damaged districts or Kebeles.

### 3.4 Discussion

Guder is the highest sediment-yielding sub-basins of the UBNR basin (Bekele et al., 2009). So, according to Bekele et al. (2009), the soil erosion rate of the Guder sub-basin is the greatest compared to other sub-basin of the Abay basin of the UBNR basin. Hurni (1993) reported a soil loss rate of 300 t/ha from cropland (Sonneveld et al., 2011), underlining the scarcity of data, prepared a tentative nationwide map of mean annual soil loss in which soil loss varied markedly, from zero in eastern and southeastern Ethiopia to >100 t ha/yr in the region including the UBNR basin. A regional sediment yield study in the UBNR basin and Atbara River as stated by (Balthazar et al., 2013) reported spatial variability between 4 and 49 t/ha, whereas Betrie et al. (2011), reported between 0 and ≥150 t/ha and the moderate class ranges between 20–70 t/ha using a SWAT model. Tiruneh and Ayalew (2016) reported an annual erosion rate greater than 100 t/ha as about 0.95% in the UBNR basin of Ethiopia. Mohammed and Asmamaw (2021) stated the soil loss ranges 117–192 ton/ha/year on a slope of >30% using RUSLE in Bahir Dar, Amhara region, UBNR basin of Ethiopia. According to Haregeweyn et al. (2017) Guder sub-basin is more off in severe erosion condition. He also reported erosion rate up to 200 t/ha/year in Abay basin of UBNR basin of Ethiopia. In this study annual erosion rate greater than 100 t/ha was only 7%. My estimates of soil erosion rate variability from the basin are in reasonable agreement with most previous studies.

In this study K-factor and R-factor of 0.07–0.16 and 514.6–672.2mm were estimated respectively. Similar soil erodibility factors and Rainfall erosivity factors were estimated in some basins of Upper Blue Nile, Ethiopia by different scholars at different watersheds (Tiruneh and Ayalew, 2016; Kebede et al., 2021) and so many others. Kebede et al. (2021) reported K-factor ranges from 0.15–0.35, and R-factor ranges 745.16–921.7mm in Beles watersheds of Upper Blue Nile, Ethiopia. Tiruneh and Ayalew (2016) reported K-factor, and R-factor of from 0.15–0.2, and 750.6 respectively at Enfraz watershed of Upper Blue Nile Ethiopia. Mohammed and Asmamaw (2021) reported a K-factor of 0.15–2.0 and R-factor of 711.5–1098.1 in Bahir Dar District of Upper Blue Nile basin, Ethiopia.

The study compared with other research the model output with different kinds of literature conducted on Upper Blue Nile Sub-basins at different spatial and temporal scales. In similar study assessed at the UBNR basin (Haregeweyn et al., 2017) reported an average annual estimated soil loss of 27.5 t ha−1 yr−1, whereas Yesuph and Dagnew (2019) predicted a mean soil erosion of 37 t ha^−1^ yr^−1^ in the Beshillo watersheds of the UBNR basin. Shiferaw (2012) estimated a mean soil loss of 30.9 tha^−1^yr^−1^ for Legemara watershed in Borena district. Bewket and Teferi (2009) assessed an average soil loss of 93 t ha^−1^ yr^−1^ for the Chemoga watershed. Similarly Ayalew, 2015 estimated a mean annual soil loss rate of around 9.1 t ha^−1^ yr^−1^ in the in the highlands of Ethiopia like Zingin watershed. In very degraded sloping regions and at specific spots of steep slopes of the watershed, soil loss rate may be ranged to 503.54 t ha^−1^ yr^−1^ as stated by (Kebede et al., 2021). As compared to the result reported by different scholars the predicted annual soil loss was generally rational. Though the quantitative soil loss estimation is unreliable in Ethiopia; several scholars strongly reported that soil erosion has endangered the UBNR basin of Ethiopia. The probable cause may be evaluation scales, the difference in time, input data, and the methods and the models applied (Hurni et al., 2008; Yesuph and Dagnew, 2019). But regarding the soil erosion severity in Ethiopia, the points stressed by different scholars have a strong similarity. Similarly, there is always spatial and temporal variation in the magnitude of average annual soil loss (Haregeweyn et al., 2017).

If this erosion rate continues, the sustainability of downstream reservoirs, including the closely completed Grand Ethiopian Renaissance Dam (GERD), will be endangered by sedimentation. Furthermore, soil erosion is also responsible for sediment transport and other nutrients, which are deposited in reservoirs and riverbed sediments. These nutrients could lead to the eutrophication of reservoir water in addition to the loss of agricultural productivity in the contributing area (Haregeweyn et al., 2008). As stated by Sonneveld (2002), and the World Bank, 2007 soil erosion caused economic loss as most of the Ethiopian population depend on agriculture. So, the output of this research is significant for creating awareness about the status of Guder watersheds to plan conservation measures for stakeholders, policymakers, government and soil and water conservation agents.

### 3.5 Limitations of the study and USLE concept to model soil erosion

The limitation of the study was the lack of measured soil loss data to validate the delineated annual soil erosion rate. Nevertheless, there was no triangulation in the study, rational results were observed with comparing to the study conducted by different scholars on other sub-basin of the Upper Blue Nile Basins of Ethiopia. As the delineated soil loss may have uncertainties, future research should target reducing uncertainties with the concept of triangulation. Comprehensive and well-planned measurement campaigns are crucially needed for soil erosion modelling validation as well as deepening the process understanding. Almost all of the modelling factors were satellite data, as the measured data are rarely available. USLE model estimates the soil loss only on the annual time scale, not on daily and monthly time scales like SWAT.

## 4 Conclusion and recommendations

In this study, GIS and RS data were applied to estimate soil erosion potential or hazard and priority classification for a fast treatment plan using a simple model USLE. The study successfully delineated the Guder watersheds' erosion potential and its classification. It has given an honestly dependable estimation of soil loss and delineation of erosion-affected areas. The result of the study would create soil and water conservation awareness and provide information for management strategies to preserve the soil from more damage that cannot be easily amended. The results certainly indicated that, despite the fact a certain degree of uncertainty and inaccuracies are existing, the USLE model was effectively utilized at the watershed with the small data inputs.

The results show that 64.3% of the watershed area undergoes moderate to very slight erosion, 19.8% high erosion, 15.8% from severe to very severe erosion, insignificant (only 0.1%) catastrophic erosion and according to Morgan classification (Morgan, 2009). As concluded from the result, soil management interventions would be exactly targeted and priority should be given to areas with severe, very severe, and catastrophic erosion. The study result was also certain with about 16.3 million tons of soil has been eroded from this study area annually.

The study was certain with an average annual soil loss of 25.23 tha^−1^yr^−1^ and the result indicated that only 7% of the Guder watersheds go beyond 100 tha^−1^yr^−1^ erosion rate. Woreda/district-wise classification shows that the middle of Steep slopes Mountainous parts of Jeldu, Ginde Beret, Ifata, Mida Kegn, Ambo, parts Ababo and Horo Guduru, Toke Kutaye, along the boundary of Midakegn and Cheliya parts found in the study area borderline, were found in severe to very severe erosion. Hence, it is recommended that this watershed needs prompt consideration from a soil management and conservation perspective.

## 5 Animal rights

This article does not contain any studies with animal subjects.

## 6 Human rights

This article does not contain any studies with human subjects performed by any of the authors.

## Declarations

### Author contribution statement

Timketa Adula Duguma: Conceived and designed the experiments; Performed the experiments; Analyzed and interpreted the data; Contributed reagents, materials, analysis tools or data; Wrote the paper.

### Funding statement

The work was supported by Ambo University, Ethiopia.

### Data availability statement

Data will be made available on request.

### Declaration of interests statement

The authors declare no conflict of interest.

### Additional information

No additional information is available for this paper.
